# The CARDIA-trial protocol: a multinational, prospective, randomized, clinical trial comparing transthoracic esophagectomy with transhiatal extended gastrectomy in adenocarcinoma of the gastroesophageal junction (GEJ) type II

**DOI:** 10.1186/s12885-020-07152-1

**Published:** 2020-08-20

**Authors:** Jessica M. Leers, Laura Knepper, Arjen van der Veen, Wolfgang Schröder, Hans Fuchs, Petra Schiller, Martin Hellmich, Ulrike Zettelmeyer, Lodewijk A. A. Brosens, Alexander Quaas, Jelle P. Ruurda, Richard van Hillegersberg, Christiane J. Bruns

**Affiliations:** 1 Department of General, Visceral, Cancer and Transplantation Surgery, University of Cologne, Kerpener Str. 62, 50937 Cologne, Germany; 2 Department of Surgical Oncology, University Medical Center Utrecht, Heidelberglaan 100, 3584 CX Utrecht, The Netherlands; 3 Institute of Medical Statistics and Computational Biology, University of Cologne, Robert-Koch-Str. 10, 50931 Cologne, Germany; 4 Clinical Trials Centre Cologne, University of Cologne, Gleueler Str. 269, 50935 Cologne, Germany; 5 Department of Pathology, University Medical Center Utrecht, Heidelberglaan 100, 3584 CX Utrecht, The Netherlands; 6 Institute for Pathology, University of Cologne, Kerpener Str. 62, 50937 Cologne, Germany

**Keywords:** esophageal adenocarcinoma, gastroesophageal junction, Siewert type II, cardia carcinoma, esophagectomy, gastrectomy

## Abstract

**Background:**

Adenocarcinoma of the gastroesophageal junction (GEJ) Siewert type II can be resected by transthoracic esophagectomy or transhiatal extended gastrectomy. Both allow for a complete tumor resection, yet there is an ongoing controversy about which surgical approach is superior with regards to quality of life, oncological outcomes and survival. While some studies suggest a better oncological outcome after transthoracic esophagectomy, others favor transhiatal extended gastrectomy for a better postoperative quality of life. To date, only retrospective studies are available, showing ambiguous results.

**Methods:**

This study is a multinational, multicenter, randomized, clinical superiority trial. Patients (*n* = 262) with a GEJ type II tumor resectable by both transthoracic esophagectomy and transhiatal extended gastrectomy will be enrolled in the trial. Type II tumors are defined as tumors with their midpoint between ≤1 cm proximal and ≤ 2 cm distal of the top of gastric folds on preoperative endoscopy. Patients will be included in one of the participating European sites and are randomized to either transthoracic esophagectomy or transhiatal extended gastrectomy. The trial is powered to show superiority for esophagectomy with regards to the primary efficacy endpoint overall survival. Key secondary endpoints are complete resection (R0), number and localization of tumor infiltrated lymph nodes at dissection, post-operative complications, disease-free survival, quality of life and cost-effectiveness. Postoperative survival and quality of life will be followed-up for 24 months after discharge. Further survival follow-up will be conducted as quarterly phone calls up to 60 months.

**Discussion:**

To date, as level 1 evidence is lacking, there is no consensus on which surgery is superior and both surgeries are used to treat GEJ type II carcinoma worldwide. The CARDIA trial is the first randomized trial to compare transthoracic esophagectomy versus transhiatal extended gastrectomy in patients with GEJ type II tumors. Several quality control measures were implemented in the protocol to ensure data reliability and increase the trial’s significance. It is hypothesized that esophagectomy allows for a higher rate of radical resections and a more complete mediastinal lymph node dissection, resulting in a longer overall survival, while still providing an acceptable quality of life and cost-effectiveness.

**Trial registration:**

The trial was registered on August 2nd 2019 at the German Clinical Trials Register under the trial-ID DRKS00016923.

## Background

**Fig. 1:**
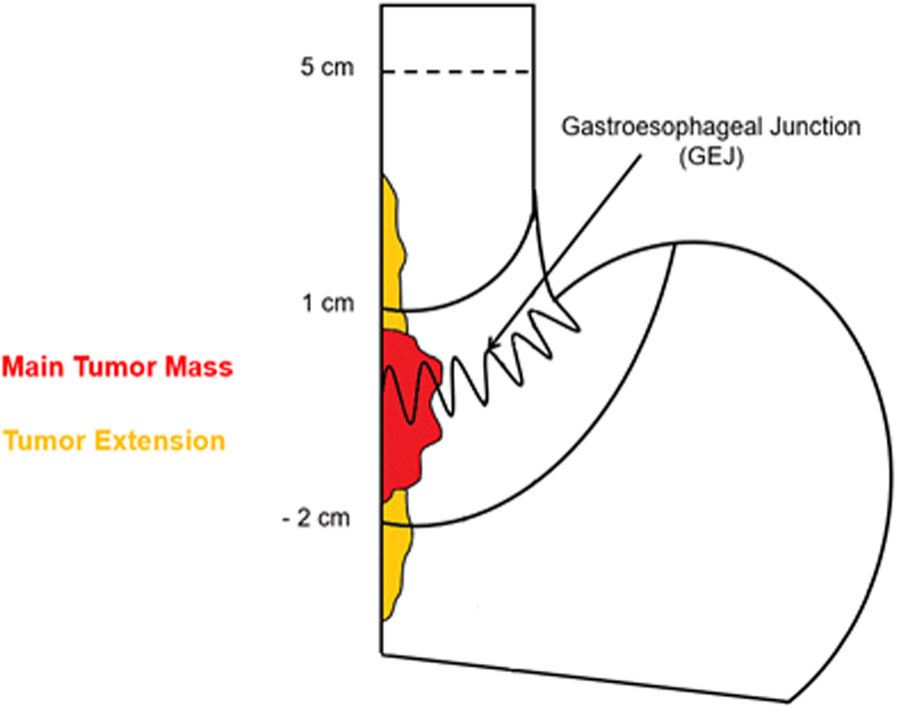
Endoscopic classification of GEJ type II tumors according to the Siewert classification of GEJ cancer. Type II tumors have their midpoint 1 cm above to 2 cm below the cardia.

Adenocarcinomas of the gastroesophageal junction (GEJ) are located at the borderline of the stomach and esophagus. GEJ tumors show an increase in incidence in the Western world. In the Netherlands for example, the incidence of esophageal cancer more than doubled in a period of less than 20 years [1]. Approximately 27% of patients with a GEJ tumor, suffer from a GEJ Siewert type II ‘true’ cardia carcinoma, meaning that the midpoint of the tumor is between ≤1 cm proximal and ≤ 2 cm distal from the GEJ (see Fig. 1) [2]. Earlier studies suggested that these tumors can have two distinct etiologies (esophageal and gastric) [3], whereas more recent genetic analysis showed that the origin may be similar [4]. Regardless of the hypothesized origin, given that the incidence of GEJ cancer has risen by up to 350% in Western Europe since the 1970’s, determination of optimal treatment is imperative to improve patient outcome [5].

Successful surgery is the cornerstone of multimodal treatment leading to a 5-year survival rate of 40–50% [6, 7]. There is an ongoing controversy about the optimal surgical approach for Siewert type II tumors. In order to provide the best, intentionally curative, treatment in patients with GEJ tumors, a radical resection of the tumor needs to be combined with removal of adjacent lymph nodes. This can be achieved by two surgical approaches: a transthoracic esophagectomy or a transhiatal extended gastrectomy. Two recent international surveys on this topic show that the worldwide preferred surgical approach for Siewert type II tumors was extended gastrectomy in 66% of respondents, followed by esophagectomy in 27% [8]. Although extended gastrectomy was found in some studies to be associated with a higher quality of life [9, 10], oncological outcome may be compromised with a higher rate of microscopic neoplastic invasion of the circumferential resection margin and less lymph node metastases resected [11]. To date, the superior surgical approach concerning survival, oncological outcome and quality of life remains unclear, as only retrospective studies are available, showing ambiguous results. This lack of level 1 evidence translates to the lack of consensus in current clinical practice worldwide.

## Methods

### Objective

The objective of this study is to compare transthoracic esophagectomy versus transhiatal extended gastrectomy in patients with resectable Siewert type II GEJ adenocarcinoma. The primary efficacy endpoint is overall survival and key secondary endpoints are complete resection (R0), post-operative complications, number and localization of tumor infiltrated lymph nodes at dissection, disease-free survival, quality of life and cost-effectiveness. It is hypothesized that esophagectomy allows for a higher rate of radical resections and a more complete mediastinal lymph node dissection resulting in a longer overall survival, while providing an acceptable quality of life and cost-effectiveness.

### Study design

This study is a multinational randomized clinical trial comparing the two surgical procedures. High-volume academic and non-academic hospitals in Germany, the Netherlands, Sweden, Ireland, Switzerland and France will participate in the trial. It is conducted in accordance with the principles of the Declaration of Helsinki and Good Clinical Practice Guidelines. The protocol has so far been approved by the independent ethics committee of the University Hospital of Cologne, University of Leipzig Medical Center, University Hospital rechts der Isar, University Medical Center of the Johannes Gutenberg University Mainz and the Medical Center – University of Freiburg. Any modifications to the protocol which may impact the trial will be communicated with the participating institutions and approved by the ethics committee again. The surgeon, patient and coordinating researcher will not be blinded for the allocated treatment.

### Study population

The study will evaluate patients with GEJ type II carcinoma whose tumor can be safely resected by both transthoracic esophagectomy and transhiatal extended gastrectomy. In detail, the inclusion criteria are:

- Histologically proven adenocarcinoma of the GEJ type II
- Resectable by both transthoracic esophagectomy and transhiatal extended gastrectomy according to the local surgical investigator
- Pre-treatment stage cT1-4a, N0–3, M0
- In case of stage cT4a, curative resectability must be explicitly verified by the local surgical investigator prior to randomization
- Completion of all four cycles of chemotherapy (FLOT) preoperatively, in case of locally advanced tumors (cT3-T4 or N+)
- Age ≥ 18
- ECOG Eastern Cooperative Oncology Group (ECOG) performance status 0–2
- ASA < 4.
- Adequate bone marrow function (white blood cells > 3 × 10^9 /l; hemoglobin > 9 g/dl; platelets > 100 × 10^9 /l), renal function (glomerular filtration rate > 60 ml/min), and liver function (total bilirubin < 1.5x upper level of normal (ULN), aspartate transaminase (AST) < 2.5x ULN and alanine transaminase (ALT) < 3x ULN)
- Written informed consent

The exclusion criteria are:

- Histologically proven adenocarcinoma of the GEJ type I and III
- Tumor resectable only by transthoracic esophagectomy or only by transhiatal extended gastrectomy, according to the local surgical investigator
- Tumor expanding more than 5 cm proximal of the GEJ
- Positive lymph nodes only resectable by transthoracic esophagectomy (i.e. in the mid-upper mediastinum) or only resectable by transhiatal extended gastrectomy according to the local surgical investigator.
- Clinically significant (active) cardiac disease (i.e. symptomatic coronary artery disease or myocardial infarction within last 12 months), resulting in a left ventricular ejection fraction < 50% (determined by echocardiography)
- Clinically significant lung disease (forced expiratory volume in one second (FEV1) < 1.5 l/s)
- Pregnant women and nursing mothers

### Patient screening and chemotherapy

**Fig. 2:**
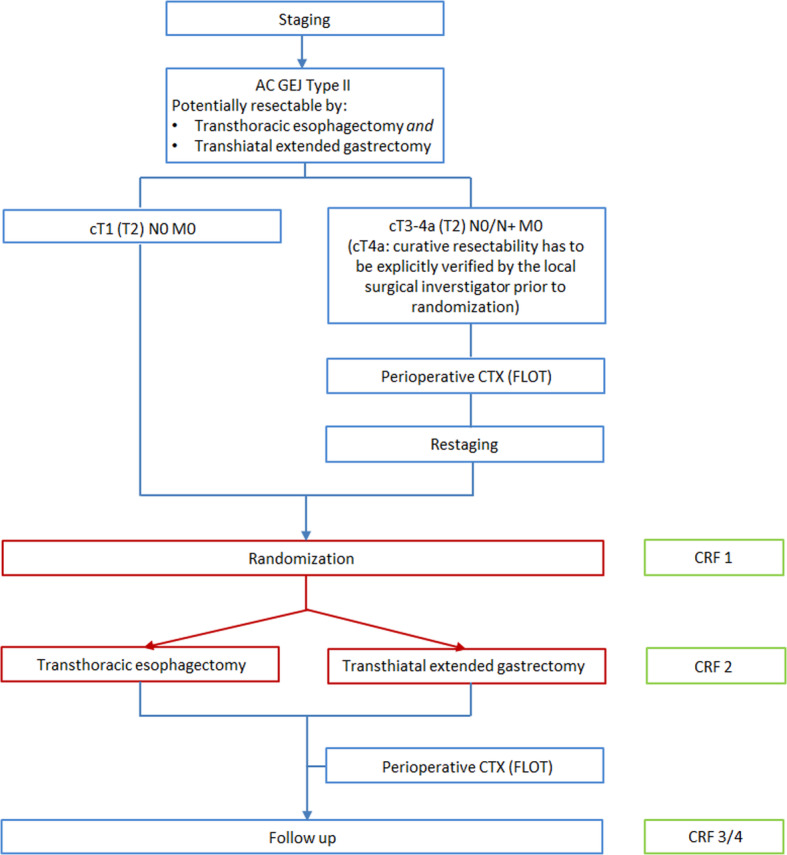
Trial flow chart.

Figure 2 displays the trial flow. All patients will undergo evaluation in a multidisciplinary tumor board and standard tumor staging before inclusion. The staging examinations will include an endoscopy with a detailed description of the tumor localization, the classification according to Siewert and biopsy extraction for histology. To standardize endoscopic definition of Siewert type II tumors, a separate Standard Operating Procedure (SOP) for staging endoscopies has been developed. Type II tumors are defined as all tumors in which the center of the main tumor mass (half the distance between the proximal and distal edges of the main tumor mass) is 1 cm above to 2 cm below the cardia. The GEJ (or cardia) is defined as the top of gastric folds after desufflation. Tumors that expand more than 5 cm above the GEJ will also be excluded from the trial, as these are not potentially resectable by a transhiatal extended gastrectomy. In the case of severe tumor stenosis, which prevents passage of the (normal and ultra slim) endoscope and therefore an endoscopic tumor classification according to Siewert, the patient can’t be included in the trial.

A computed tomography of the thorax and abdomen will be performed to identify metastatic disease and the extension of the disease. In addition, endoscopic ultrasound to determine the tumor infiltration depth and fluorodeoxyglucose positron emission tomography with CT (FDG-PET/CT or PET) to identify distant metastases are recommended in staging, yet not mandatory. The complete preoperative work-up will further include a physical examination, medical history, demography, vital signs and laboratory tests. In patients with a history or symptoms of cardiac and/or pulmonary disease, additional cardiology consultation, echocardiography (ejection fraction > 50%) and pulmonary function tests (FEV1 > 1.5 l) will be mandatory.

For patients with locally advanced adenocarcinomas (T2 or higher, N+) multimodal treatment is the standard care according to international guidelines [12]. For T3-T4 or N+ staged patients, perioperative chemotherapy according to the FLOT protocol [13] will be obligatory for patients in the CARDIA trial. For T2 N0 staged patients, perioperative FLOT chemotherapy will be recommended, but will not be compulsory for inclusion in the CARDIA trial. It can be administered based upon the individual decision per patient. T1 N0 M0 staged patients will receive a primary resection. Perioperative chemotherapy is not part of the investigated treatment and thus does not have to be performed at the designated trial sites. If patients with locally advanced adenocarcinoma GEJ type II were fully diagnosed as described above by long-term reliable external gastroenterological collaborators of high-volume centers and secondly referred to the respective surgical department after finishing multimodal treatment with the FLOT regimen, they can also be included in the CARDIA trial.

For patients undergoing chemotherapy, restaging after four preoperative cycles will be mandatory and screening examinations will take place during restaging. Patients who do not undergo chemotherapy will receive all screening examinations during their primary staging.

### Patient inclusion and randomization

When baseline assessments and staging or restaging are completed, inclusion and exclusion criteria for the trial will be validated. Written informed consent will be obtained from all trial participants. Immediately after the patient has given his written consent to participate in the trial, randomization to one of the therapeutic arms will be performed by means of a 24/7 internet service based on permuted blocks of varying length, stratified by trial site and/or surgeon and tumor stage.

### Patient follow-up

The follow-up will include seven regular visits for check-up every three to six months with a total of 24 month after discharge. Further follow-up will be conducted as quarterly phone calls to determine survival up to 60 months after discharge. For a detailed listing of the procedures and items recorded in the eCRF for each trial visit, see Table 1. If a tumor recurrence is clinically suspected during the follow-up period, a CT of the thorax and abdomen as well as an endoscopy will be performed on indication to further document the disease-free survival. Loss-to-follow-up will be minimised by commissioning a person at the trial site to manage and encourage follow-up and providing of excellent and free medical care. We therefore assume a small attrition rate of 0.05 per year and study arm.

V0 Screening, V1 Baseline/Randomization, V2 Discharge, V3 + 1 month, V4 + 3 months, V5 + 6 months, V6 + 9 months, V7 + 12 months, V8 + 18 months, V9 + 24 months, Survival Follow-up up to 60 months – quarterly by phone. ^1^Negative serum pregnancy test during screening period for women of child-bearing age. ^2^Only in case of a clinically suspected tumor recurrence.

**Table 1:** Visit schedule

	**V0**	**V1**	**V2**	**V3**	**V4**	**V5**	**V6**	**V7**	**V8**	**V9**	**Survival-** **Follow-up**
Demography	X										
Medical history	X										
Oncological history	X										
Tumor classification	X										
Pregnancy test^1^		X									
Inclusion / Exclusion		X									
Laboratory	X										
Biopsy	X										
Randomisation		X									
Physical examination	X		X	X	X	X	X	X	X	X	
Anamnesis	X			X	X	X	X	X	X	X	
Endoscopy	X				X^2^	X^2^	X^2^	X^2^	X^2^	X^2^	
CT/MRI	X				X^2^	X^2^	X^2^	X^2^	X^2^	X^2^	
Concomitant Medication	X		X	X	X	X	X	X	X	X	
EORTC QLQ-C30, −STO22, −OG 25-		X	X		X	X	X	X	X	X	
OP – Description			X								
Post-OP Complication			X								
Pathology			X								
Reference pathology			X								
AE/SAE			X	X	X						
Survival				X	X	X	X	X	X	X	X
EOS											or earlier for premature withdrawal

### Surgery

Patients will either receive a transthoracic esophagectomy or transhiatal extended gastrectomy depending on randomization outcome. However, the surgeon should change his/her surgical strategy if tumor-free resection margins can’t be achieved, as described below. In general, technical details of both surgical procedures are left to the individual surgeon’s preference as long as the primary goal of a complete tumor resection is achieved. This also includes all established minimally invasive surgical techniques as well as the application or support of robotic surgery devices.

### Transthoracic esophagectomy

**Fig. 3:**
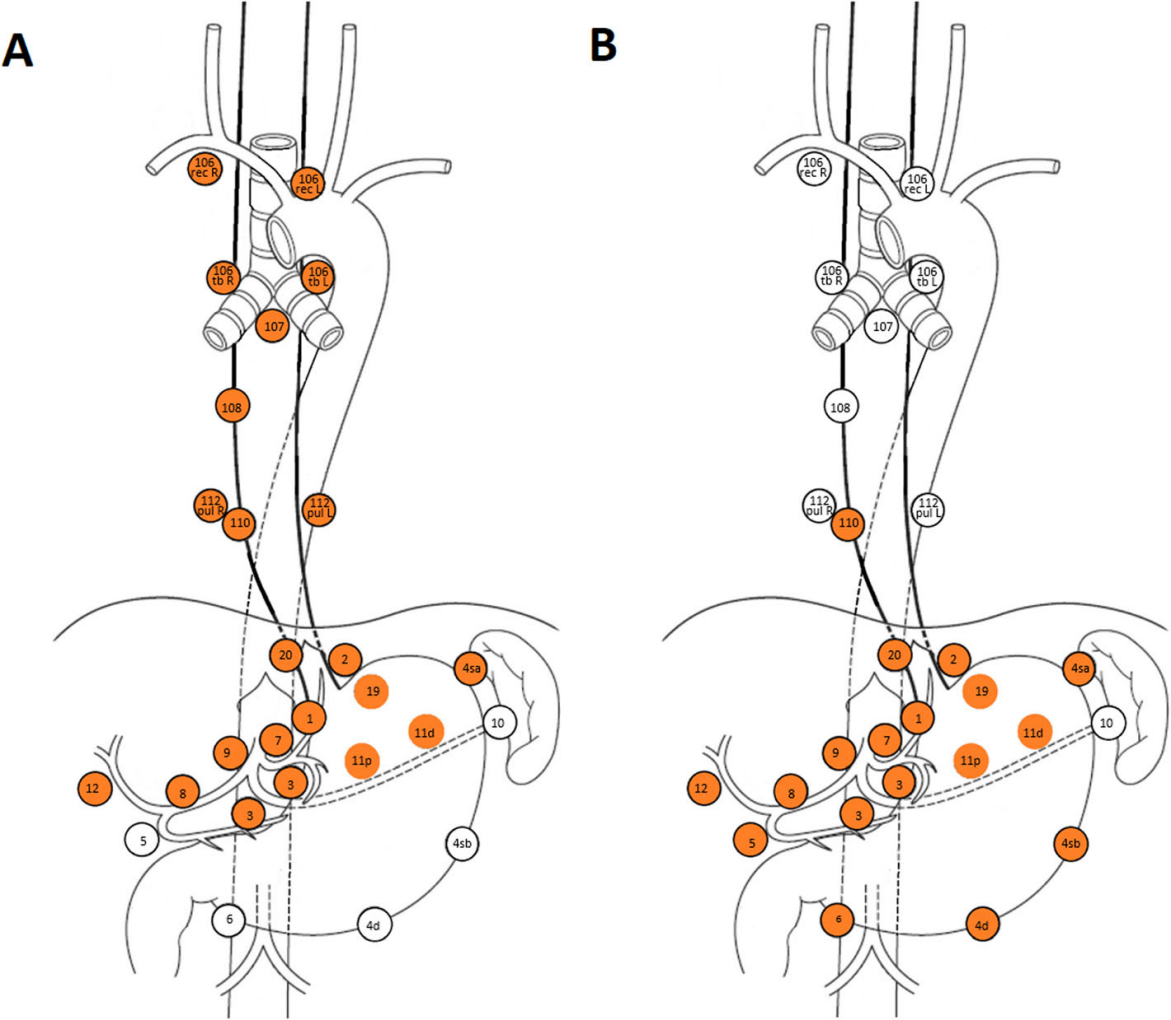
Obligatory and optional lymph node stations for lymph node dissection during transthoracic esophagectomy (A) and transhiatal extended gastrectomy (B). Lymph node stations which should be resected are marked in orange [15]., altered.

The esophagectomy will be performed by means of a right transthoracic approach combined with resection of the proximal stomach (GEJ). Transhiatal access or access by thoraco-phreno-laparotomy is not allowed. A gastric conduit is constructed, and continuity is re-established by intrathoracic anastomosis (Ivor-Lewis). Cervical anastomosis (McKeown) is not allowed. The procedure includes a 2-field lymphadenectomy, resection of lymph node stations 1–3, 4sa, 7, 8a, 9, 11p, 11d, 12, 106 tb R, 106 rec R, 107, 108, 109 and 110 according to JGCA/JES [14, 15] is mandatory (see Fig. 3). Resection of lymph node station 106 tb L and 106 rec L are recommended, yet not mandatory (see appendix, Table 1). A total of at least 25 lymph nodes should be examined per resection according to the German S3 guidelines for gastric cancer [16]. In addition to the lymph node stations, the thoracic duct compartment also has to be resected [17].

### Transhiatal extended gastrectomy

Transhiatal extended gastrectomy will be performed according to common practice at the specific hospitals. Reconstruction will be achieved by esophagojejunostomy and Jejunojejunostomy (Roux-en-Y reconstruction). For all patients randomized for transhiatal extended gastrectomy an intraoperative endoscopy is mandatory prior to transection of the tubular esophagus to ensure a tumor-free upper resection margin. In addition, an intraoperative frozen section of the oral resection margin should be performed to confirm a complete resection of the primary tumor. If a complete resection cannot be achieved, strategy must be changed and a transthoracic esophagectomy must be performed. Again, a 2-field lymphadenectomy is performed harvesting at least 25 lymph nodes, including lymph node stations 1–1-7, 8a, 9, 11p, 11d, 12a and the lower paraesophageal lymph node station 110 (see Fig. 3).

### Surgical quality control

The participating sites will be expert high-volume hospitals with a caseload of at least 15 esophagectomies and 10 transhiatal extended gastrectomies per year over the last 3 years. Only surgeons that are experienced to perform both types of surgery will be eligible to participate in this randomized trial. Surgeons who wish to use a total minimally invasive or robotic assisted approach for the esophagectomy or transhiatal gastrectomy must demonstrate that they have performed this surgical technique at least twenty times in a designated surgical team. This is to ensure quality of surgery and standardized perioperative management and to ensure a substantial allocation of patients to the trial within the designated recruitment period.

As an ongoing surgical quality assurance throughout the trial, photographs of the operating area will be taken during each surgery, showing the completeness of the lymphadenectomy. These images will be used for ongoing feedback during the course of the trial on the surgical technique. Additionally, they will be analyzed in depth if the half-yearly safety analysis by the Data Monitoring Committee (DMC) shows a reduced number of resected lymph nodes or high number of R1 resections, which could indicate insufficient surgical quality at the respective trial site.

### Pathological quality control

To ensure quality of the pathological examination, the lymph node dissection will be divided by the surgeon in single packages for each lymph node station so that the pathologist can individually analyze the different lymph node packages. However, the peritumoral stations will be marked en-bloc by the surgeon, instead of being resected in single packages, so analysis of the circumferential resection margin is not compromised. The pathological analysis of the entire surgical specimen will be carried out at each site according to a standard CARDIA trial pathology protocol. To ensure the quality of the pathological assessment of the circumferential resection margin, the resection margin samples of the respective surgical specimen will be re-analyzed by one of two reference pathologies which are located at the University Hospital of Cologne and the University Medical Center Utrecht.

### Postoperative treatment

The postoperative treatment will not differ between both treatment arms. Epidural analgesia will be performed, and other analgesia can be given according to each centers standard practice. Mobilization under supervision of a physiotherapist will start as soon as the patient is stable. Enteral feeding via a jejunostomy catheter for patients will be allowed. Patients will be discharged according to standard practice of each surgical center.

### Outcome measures

The primary endpoint is overall survival, which will be followed up until 60 months after discharge of surgery. The key secondary endpoints are complete resection (R0), post-operative complications according to the Esophageal Complications Consensus Group (ECCG) definitions [18] and scored on severity by the the Clavien-Dindo classification [19], number and localization of tumor infiltrated lymph nodes at dissection, disease-free survival, quality of life (EORTC QLQ-C30, QLQ-STO22, and QLQ-OG25 after 3, 6, 9, 12, 18, 24 months) and cost-effectiveness.

After consolidation of the EORTC questionnaires data during the course of the trail, a cost effectiveness analysis of both procedures based on direct costs and resulting QALYs will be performed and the incremental cost-effectiveness ratio will be calculated. For simplification of the process, only German sites classified as excellence centers will be included into the analysis, so that consistent national costs (e.g. DRG based, medical supply, labor costs based on tariffs) can be comparable.

### Data management and safety

The trial database will be developed and validated before data entry based on standard operating procedures at the Clinical Trial Centre Cologne (CTCC). All changes made to the data will be documented in an audit trail. The database will be integrated into a general IT infrastructure and safety concept with a firewall and backup system. Plausibility checks will be run during data entry. The CTCC Data Management will conduct further checks for completeness and plausibility and will clarify any questions with the trial sites electronically via the trial software.

The trial sites will be monitored closely to ensure patient’s safety and the quality of the data collected. Postoperative complications classified as Dindo-Clavien grade IIIb and higher (requiring surgical, endoscopic or radiological intervention under general anesthesia) or anastomosis incufficiency will be considered adverse events. A serious adverse event is defined as the death of a patient. AEs and SAEs will only be reported until three months after discharge (V4).

### Sample size calculation

The primary endpoint is overall survival. We assume a cumulative survival after 2.5 years of 45% for transthoracic esophagectomy and 30% for transhiatal extended gastrectomy (see Fig. 1c in [20]). This corresponds to a hazard ratio of 0.663. A total of 188 events need to be observed to detect this difference with the stratified log-rank test (two-sided type I error 5%, power 80%, accounting for the interim analysis according to two-sided symmetric O’Brien-Fleming type alpha-spending at 94 events). Thus, 262 patients need to be enrolled and followed up (assuming 2,5 years of accrural, 5 years of follow-up and 5% censoring per year) [21]. For the key secondary outcome R0 resection Parry et al. report percentages of 87 and 71%, respectively [20]. This corresponds to an odds ratio of 2.73. Thus, 262 enrolled patients are also sufficient to give the stratified Mantel-Hanszel test at least 80% power (two-sided type I error 5%). Before participating in the trial, each center reported possible recruitment figures based on the number of patients with GEJ type II tumors in recent years. Screening will continue until the target sample size is achieved.

### Statistical analysis

The primary analysis will be performed on the full analysis set (intention-to-treat) and comprises all patients with written informed consent and who were operated. The analysis is as assigned by stratified randomization (i.e. site or surgeon and tumor stage). The primary outcome of overall survival will be evaluated by the stratified log-rank test and is event-driven (i.e. following observation of 188 events). The hazard ratio with 95% confidence interval (CI) is estimated by stratified Cox regression. The assumption of non-informative censoring is examined using standard approaches (i.e. Kaplan-Meier curves, examining patterns of censoring across covariates and association between censoring and covariates). The key secondary outcome R0 resection rate is analyzed by comparing the corresponding odds between treatment groups with the stratified Mantel-Haenszel test. A missing assessment is counted as failure. Contingency tables of strata with “empty rows or columns” are pooled. The combined Mantel-Haenszel odds ratio with 95% CI is calculated. The potential impact of clustering associated with both interventions is addressed in a sensitivity analysis [22, 23]. The secondary outcome quality of life is averaged over 36 months (area under curve, AUC) and tested for non-inferiority of the esophagectomy arm regarding a margin of 80%. Missing values are linearly interpolated. The key outcomes overall survival, R0 resection and quality of life are tested in a fixed sequence, thus no alpha-correction is required for strong type I error control (5%, two-sided).

Subgroup analyses are done by sex, tumor stage and (essential) compliance with the protocol (i.e. per protocol analysis). Adverse events are summarized and listed by seriousness, severity and relatedness to treatment. Cumulative survival 30, 36 and 60 months following surgery is estimated by Kaplan-Meier method (with 95% CI according to Greenwood’s formula). Methods for rates and proportions are used to describe hospital mortality and the incidence of post-operative complications. A linear mixed model is fit to health related quality of life data from EORTC questionnaires QLQ-C30, QLQ-STO22, and QLQ-OG25. Cost-effectiveness is determined as the ratio ∆C/∆E where ∆C is the difference in resource use (costs) and ∆E the gain in months of life or in quality of life. The cost-effectiveness (cost per life-month gained or cost per quality-of-life-unit) will be calculated from a societal perspective.

### Interim analysis

When half the events (94 deaths) have occurred, an interim analysis will be performed (O’Brien-Fleming type alpha-spending). The outcomes will be evaluated by the DMC. Premature termination of the trial will be considered if the interim analysis or other research show a significant difference in survival between both treatment groups. In addition, the stop-criteria are: < 70% complete resection without tumor residual (R0) in one or both arms as well as > 50% less than 10 lymph nodes harvested in one or both arms. These criteria will be continuously evaluated by the DMC.

## Discussion

In the year 2000, a study on Siewert type I-III GEJ tumors was conducted that compared both surgical approaches. No difference was found between esophagectomy and extended gastrectomy regarding 30-day mortality and 5-year survival [6]. Two years later a retrospective study was conducted in which no statistically significant survival benefit for either one of the surgical approaches was found [11]. Post-operative mortality turned out to be independent of surgical procedure. However, extended gastrectomy was associated with higher microscopic neoplastic invasion of the resection margin than esophagectomy (R0 resection rate: 23.7 and 6.6%, respectively). No differentiation between Type I, II or III was made. Another retrospective research on GEJ type I-III tumors showed that there was no significant difference found in R0 resection, lymph node removal, or post-operative mortality rates with respect to operative approaches. However, gastrectomy was demonstrated to have a significantly worse 5-year survival than esophagectomy, 27 and 37% respectively [24]. In 2014, the Haverkamp et al. systematically reviewed manuscripts published between 1995 and 2013 on surgical strategies of adenocarcinomas of the GEJ and found no clear oncological benefit of either esophagectomy or gastrectomy [10]. More recently, Dutch data suggested that in patients with a type II GEJ adenocarcinoma, a positive circumferential resection margin was more common with gastrectomy. Furthermore, the high prevalence of mediastinal nodal involvement indicates that a full lymphadenectomy of these lymph node positions should be considered. However, no significant difference in 5-year survival was found [10, 20, 25].

The systematic review from Haverkamp et al. included 2 retrospective studies on quality of life, these studies suggested a better quality of life after gastrectomy [10]. The Cologne group recently published a retrospective monocentric analysis of own data on long-term quality of life after surgery for adenocarcinoma of the esophagogastric junction type II and detected that health related quality of life after extended gastrectomy with Roux-en-Y reconstruction was indeed superior to that after esophagectomy and gastric tube reconstruction. Patients with cancer-free survival of at least 24 months after esophagectomy or extended gastrectomy for GEJ type II were identified from a prospectively maintained database and EORTC questionnaires were send to these patients. Improved HRQL after gastrectomy was mainly due to less pulmonary symptoms perioperatively and reflux-related symptoms in the long-term follow-up [9].

To date, only retrospective studies are available on surgical therapy of GEJ type II carcinoma, leading to a great controversy about the superior surgical procedure [24]. The CARDIA trial will be the first randomized, clinical trial to determine the surgery of choice for this disease An essential prerequisite for a realistic statement on the therapy of GEJ II tumors is the exact definition of these tumors according to the Siewert classification, which has been described as challenging by other studies [25, 26]. Therefore, all sites will use the endoscopic SOPs in which this is defined in detail. Not only the spread of the main tumor mass but also the tumor extension are considered to ensure resectability of the tumors preoperatively. As perioperative chemotherapy is standard care for all T3–4 or N+ stage patients according to international guidelines [12], chemotherapy is also obligatory for these patients in the current trial. By including patients after chemotherapy, the patient cohort represents the actual group of patients with GEJ II tumors, of which up to 60% are already in an advanced tumor stage at initial diagnosis that requires neoadjuvant therapy [27]. The stratification of randomization is intended to reduce the regional influence of the site or the surgeon on the one hand, and the influence of the tumor stage on the primary outcome on the other.

Several quality control measures were implemented through every step of the protocol to improve data reliability and therefore the significance of this trial, as the quality of surgery has often been the subject of discussion in clinical gastroesophageal cancer trials. Therefore, only expert high-volume hospitals with a caseload of at least 15 esophagectomies and 10 transhiatal extended gastrectomies per year over the last 3 years are eligible to participate in the trial. Training materials will be provided to all sites before the start of the trial. During the course of the trial, photographs will be taken by the surgeons during each operation, to show the completeness of the lymphadenectomy. They are evaluated by the medical coordinators of the trial and used for continuous feedback to the individual sites. In addition, the DMC can also view the images if there are indications for insufficient surgical quality at an individual site. To ensure not only the surgical but also the pathological quality of the trial, all obligatory lymph node stations are clearly defined and will be sent to the pathology department in separate packages. In addition, a reference pathology was established for the assessment of the resection margins at the UCC and UMCU.

In conclusion, the incidence of GEJ cancer markedly increases. All studies that investigated both surgical procedures only consisted of retrospective series showing ambiguous results on survival and postoperative morbidity.

The CARDIA trial is the first randomized, clinicaltrial that compares transthoracic esophagectomy versus transhiatal extended gastrectomy in patients with GEJ type II tumors. To ensure the data reliability and trial quality, several control measures were implemented in the trial protocol. We hypothesize that transthoracic esophagectomy will allow for a higher rate of radical resections and a more complete mediastinal lymph node dissection, resulting in a longer overall survival, while providing an acceptable quality of life and cost-effectiveness.

### Trial status

Pre-selection visits were conducted at sixteen potential study centers of which twelve were selected for inclusion. The trial centers in Cologne, Leipzig and Munich have been initiated. In addition, the study protocol was approved by two further ethics committees. Recruitment has already started at the University Clinic Cologne, where four patients have been enrolled so far (status May 09th 2020).

## Supplementary information

**Additional file 1.** The CARDIA trial protocol_appendix. Table 1: Lymphadenectomy. Table of lymph node stations that have to be resected during transthoracic esophagectomy and transhiatal extended gastrectomy, including a definition of each station by the Japan Esophageal Society [15] and Rice et al. [28].

## Data Availability

Data sharing is not applicable to this article as no datasets were generated or analysed until this point in the study. The final dataset will be available for the sponsor, the statistiscan, the data management team, and the DMC. The trial interim and final results will be published in a scientific journal. All trial data will be published on behalf of the CARDIA study group, consisting of at least two memebers of every study center. The full protocol and statistical code will not be publicly accessable.

## Abbreviations

AE: Adverse Event
AUC: Area Under the Curve
CT: Computerised Tomography
CRF: Case Report Form
DMC: Data Monitoring Committee
ECCG: Esophageal Complications Consensus Group
ECOG: Eastern Cooperative Oncology Group
EORTC: European Organization for Research and Treatment of Cancer
EOS: End of Study
FEV: Forced Expiratory Volume
GEJ: Gastro-esophageal Junction
HRQL: Health Related Quality of Life
MRI: Magnetic Resonance Imaging
PET: Positron emission tomography
SAE: Serious Adverse Event
ULN: Upper Level of Normal

## References

[1] Nederlandse Kankerregistratie. Cijfers over kanker. 2015. http://www.cijfersoverkanker.nl/. Accessed June, 2016.

[2] Siewert JR, Stein HJ (1998). Classification of adenocarcinoma of the oesophagogastric junction. Br J Surg..

[3] McColl KE, Going JJ (2010). Aetiology and classification of adenocarcinoma of the gastro-oesophageal junction/cardia. Gut..

[4] Cancer Genome Atlas Research Network; Analysis Working Group: Asan University; BC Cancer Agency; Brigham and Women’s Hospital; Broad Institute; Brown University; Case Western Reserve University; Dana-Farber Cancer Institute; Duke University; Greater Poland Cancer Centre; Harvard Medical School; Institute for Systems Biology; KU Leuven; Mayo Clinic; Memorial Sloan Kettering Cancer Center; National Cancer Institute; Nationwide Children’s Hospital; Stanford University; University of Alabama; University of Michigan; University of North Carolina; University of Pittsburgh; University of Rochester; University of Southern California; University of Texas MD Anderson Cancer Center; University of Washington; Van Andel Research Institute; Vanderbilt University; Washington University; Genome Sequencing Center: Broad Institute; Washington University in St. Louis; Genome Characterization Centers: BC Cancer Agency; Broad Institute; Harvard Medical School; Sidney Kimmel Comprehensive Cancer Center at Johns Hopkins University; University of North Carolina; University of Southern California Epigenome Center; University of Texas MD Anderson Cancer Center; Van Andel Research Institute; Genome Data Analysis Centers: Broad Institute; Brown University; Harvard Medical School; Institute for Systems Biology; Memorial Sloan Kettering Cancer Center; University of California Santa Cruz; University of Texas MD Anderson Cancer Center; Biospecimen Core Resource: International Genomics Consortium; Research Institute at Nationwide Children’s Hospital; Tissue Source Sites: Analytic Biologic Services; Asan Medical Center; Asterand Bioscience; Barretos Cancer Hospital; BioreclamationIVT; Botkin Municipal Clinic; Chonnam National University Medical School; Christiana Care Health System; Cureline; Duke University; Emory University; Erasmus University; Indiana University School of Medicine; Institute of Oncology of Moldova; International Genomics Consortium; Invidumed; Israelitisches Krankenhaus Hamburg; Keimyung University School of Medicine; Memorial Sloan Kettering Cancer Center; National Cancer Center Goyang; Ontario Tumour Bank; Peter MacCallum Cancer Centre; Pusan National University Medical School; Ribeirão Preto Medical School; St. Joseph’s Hospital &Medical Center; St. Petersburg Academic University; Tayside Tissue Bank; University of Dundee; University of Kansas Medical Center; University of Michigan; University of North Carolina at Chapel Hill; University of Pittsburgh School of Medicine; University of Texas MD Anderson Cancer Center; Disease Working Group: Duke University; Memorial Sloan Kettering Cancer Center; National Cancer Institute; University of Texas MD Anderson Cancer Center; Yonsei University College of Medicine; Data Coordination Center: CSRA Inc.; Project Team: National Institutes of Health. Integrated genomic characterization of oesophageal carcinoma. Nature. 2017;541(7636):169–175. 10.1038/nature20805 PMC5651175 28052061

[5] Botterweck AA, Schouten LJ, Volovics A, Dorant E, van Den Brandt PA (2000). Trends in incidence of adenocarcinoma of the oesophagus and gastric cardia in ten European countries. Int J Epidemiol..

[6] Siewert JR, Feith M, Werner M, Stein HJ (2000). Adenocarcinoma of the esophagogastric junction: results of surgical therapy based on anatomical/topographic classification in 1002 consecutive patients. Ann Surg..

[7] An JY, Baik YH, Choi MG, Noh JH, Sohn TS, Bae JM (2010). The prognosis of gastric cardia cancer after R0 resection. Am J Surg..

[8] Haverkamp L, Seesing MF, Ruurda JP, Boone J, Hillegersberg RV (2017). Worldwide trends in surgical techniques in the treatment of esophageal and gastroesophageal junction cancer. Dis Esophagus..

[9] Fuchs H, Hölscher AH, Leers J, Bludau M, Brinkmann A, Schröder W, Alakus H, Mönig S, Gutschow C (2016). Long term quality of life for surgery for adenocarcinoma of the gastroesophageal junction: extended gastrectomy or tranthoracic esophagectomy. Gastric Cancer..

[10] Haverkamp L, Ruurda JP, van Leeuwen MS, Siersema PD, van Hillegersberg R (2014). Systematic review of the surgical strategies of adenocarcinomas of the gastroesophageal junction. Surg Oncol..

[11] Mariette C, Castel B, Toursel H, Fabre S, Balon JM, Triboulet JP (2002). Surgical management of and long-term survival after adenocarcinoma of the cardia. Br J Surg..

[12] Lordick F, Mariette C, Haustermans K, Obermannová R (2016). Arnold D; ESMO Guidelines Committee. Oesophageal cancer: ESMO Clinical Practice Guidelines for diagnosis, treatment and follow-up. Ann Oncol..

[13] Al-Batran SE, Hofheinz RD, Tannapfel A (2016). Histopathologic regression after neoadjuvant docetaxel, oxaliplatin, fluorouracil, and leucoverin versus epirubicin, cisplatin, and fluorouracil or capecitabine in patients with resectable gastric or gastro-oesophageal junction adenocarcinoma (FLOT4-AIO): results from the phase 2 part from a multicenter, open label, randomized phase 2/3 trial. Lancet Oncol..

[14] Sano T, Kodera Y (2011). Japanese classification of gastric carcinoma: 3rd English edition. Gastric Cancer..

[15] Japan Esophageal Society (2017). Japanese Classification of Esophageal Cancer, 11th Edition: part I. Esophagus..

[16] [German S3-guideline “Diagnosis and treatment of esophagogastric cancer”]. Moehler M, Al-Batran SE, Andus T, Anthuber M, Arends J, Arnold D, et al. AWMF; AWMF. Z Gastroenterol. 2011;49(4):461–531. 10.1055/s-0031-1273201 21476183

[17] Schurink B, Defize IL, Mazza E, Ruurda JP, Brosens LAA, Roeling TAP (2018). Two-Field Lymphadenectomy During Esophagectomy: The Presence of Thoracic Duct Lymph Nodes. Ann Thorac Surg..

[18] Low DE, Alderson D, Cecconello I, Chang AC, Darling GE, XB DJ, Griffin SM, Hölscher AH, Hofstetter WL, Jobe BA, Kitagawa Y, Kucharczuk JC, Law SY, Lerut TE, Maynard N, Pera M, Peters JH, Pramesh CS, Reynolds JV, Smithers BM, van Lanschot JJ (2015). International Consensus on Standardization of Data Collection for Complications Associated With Esophagectomy: Esophagectomy Complications Consensus Group (ECCG). Ann Surg..

[19] Dindo D, Demartines N, Clavien PA (2004). Classification of surgical complications. A new proposal with evaluation in a cohort of 6336 patients and a survey. Ann Surg..

[20] Parry K, Haverkamp L, Bruijnen RC, Siersema PD, Ruurda JP, van Hillegersberg R (2015). Surgical treatment of adenocarcinomas of the gastro-esophageal junction. Ann Surg Oncol..

[21] Lachin JM, Foulkes MA (1986). Evaluation of sample size and power for analyses of survival with allowance for nonuniform patient entry, losses to follow-up, noncompliance, and stratification. Biometrics..

[22] Boutron I, Moher D, Altman DG, Schulz KF, Ravaud P, Group C (2008). Extending the CONSORT statement to randomized trials of nonpharmacologic treatment: explanation and elaboration. Annals of internal medicine..

[23] Papachristofi O, Klein A, Sharples L (2016). Evaluation of the effects of multiple providers in complex surgical interventions. Statistics in medicine..

[24] Barbour AP, Rizk NP, Gonen M, Tang L, Bains MS, Rusch VW, et al. Adenocarcinoma of the gastroesophageal junction: influence of esophageal resection margin and operative approach on outcome. Ann Surg. 2007;246(1):1–8. 10.1097/01.sla.0000255563.65157.d2 PMC1899203 17592282

[25] Leers JM, DeMeester SR, Chan N, Ayazi S, Oezcelik A, Abate E, Banki F, Lipham JC, Hagen JA, DeMeester TR. Clinical characteristics, biologic behavior, and survival after esophagectomy are similar for adenocarcinoma of the gastroesophageal junction and the distal esophagus. J Thorac Cardiovasc Surg. 2009;138(3):594-602. 10.1016/j.jtcvs.2009.05.039 19698841

[26] C. Pedrazzani, M. Bernini, S. Giacopuzzi, Pugliese R, Catalano F, Festini M, Rodella L, de Manzoni G. Evaluation of Siewert classification in gastro-esophageal junction adenocarcinoma: what is the role of endoscopic ultrasonography? J Surg Oncol. 2005;91(4):226–231. 10.1002/jso.20302 16121346

[27] Parry K, Haverkamp L, Bruijnen RC, Siersema PD, Offerhaus GJ, Ruurda JP, van Hillegersberg R. Staging of adenocarcinoma of the gastroesophageal junction. Eur J Surg Oncol. 2016;42(3):400–6. 10.1016/j.ejso.2015.11.014 26777127

[28] Rice TW, Patil DT, Blackstone EH. 8th edition AJCC/UICC staging of cancers of the esophagus and esophagogastric junction: application to clinical practice. Ann Cardiothorac Surg. 2017;6(2):119–30. 10.21037/acs.2017.03.14 PMC5387145 28447000

