# Supplementary material for: The CARDIA-trial protocol: a multinational, prospective, randomized, clinical trial comparing transthoracic esophagectomy with transhiatal extended gastrectomy in adenocarcinoma of the gastroesophageal junction (GEJ) type II
Source: BMC Cancer. 2020 Aug 20;20:781. doi: 10.1186/s12885-020-07152-1 (PMC7439687; doi:10.1186/s12885-020-07152-1)
Supplement: Supplementary file 1 — Additional file 1. The CARDIA trial protocol_appendix. Table 1: Lymphadenectomy. Table of lymph node stations that have to be resected during transthoracic esophagectomy and transhiatal extended gastrectomy, including a definition of each station by the Japan Esophageal Society [15] and Rice et al. [28]. [file 12885_2020_7152_MOESM1_ESM.docx]

**Appendix**

**Table 1:** Lymphadenectomy.

| **TTE** | **THG** | **Name of station** | **Definition [14, 15]** | **JGCA 3rd /JES 11th [14, 15]** | **AJCC/ UICC 8th [29]** |
| --- | --- | --- | --- | --- | --- |
|  |  |  |  |  |  |
|  |  | ***Abdominal*** |  |  |  |
| M | M | Right paracardial | Right paracardial LNs, including those along the first branch of the ascending limb of the left gastric artery | 1 | - |
| M | M | Left paracardial | Left paracardial LNs including those along the esophagocardiac branch of the left subphrenic artery | 2 | - |
| M | M | Lesser curvature | Lesser curvature LNs along the branches of the left gastric artery and along the 2nd branch and distal part of the right gastric artery | 3 | - |
| M | M | Short gastric vessels | Left greater curvature LNs along the short gastric arteries (perigastric area) | 4sa | - |
|  | M | Left gastroepiploic artery | Left greater curvature LNs along the left gastroepiploic artery (perigastric area) | 4sb | - |
|  | M | Right gastroepiploic artery | Right greater curvature LNs along the 2nd branch and distal part of the right gastroepiploic artery | 4d | - |
|  | M | Suprapyloric | Suprapyloric LNs along the 1st branch and proximal part of the right gastric artery | 5 | - |
|  | M | Infrapyloric | Infrapyloric LNs along the first branch and proximal part of the right gastroepiploic artery down to the confluence of the right gastroepiploic vein and the anterior superior pancreatoduodenal vein | 6 | - |
| M | M | Left gastric artery | LNs along the trunk of left gastric artery between its root and the origin of its ascending branch | 7 | - |
| M | M | Common hepatic artery (anterosuperior) | Anterosuperior LNs along the common hepatic artery | 8a | - |
| M | M | Celiac artery | Celiac artery LNs | 9 | - |
| M | M | Proximal splenic artery | Proximal splenic artery LNs from its origin to halfway between its origin and the pancreatic tail end | 11p | - |
| M | M | Distal splenic artery | Distal splenic artery LNs from halfway between its origin and the pancreatic tail end to the end of pancreatic tail | 11d | - |
| M | M | Hepatoduodenal ligament | Hepatoduodenal ligament LNs along the proper hepatic artery, in the caudal half between the confluence of the right and left hepatic ducts and the upper border of the pancreas | 12a | - |
|  |  | ***Thoracic*** |  |  |  |
| M |  | Thoracic duct compartment | The thoracic duct is located within the posterior mediastinum, in the paraaortic compartment. This compartment is bounded by the aortoesophageal and aortopulmonary ligament anteriorly and by the spine posteriorly [17] | - | - |
| M |  | Pulmonary ligament | Lymph nodes located in the pulmonary ligament(s), including lymph nodes adjacent to the pericardium and the inferior pulmonary vein. A distinction between left and right must be included | 112pul | 9 |
| M | M | Lower paraesophageal | Lymph nodes located around the lower thoracic esophagus | 110 | 8L/8R |
| M |  | Right main bronchus | Lymph nodes located caudal to the carina. The lateral boundaries are the right main bronchus and the extended line of the right margin of the trachea. | 109 R |  |
| M |  | Left main bronchus | Lymph nodes located caudal to the carina of the trachea. The lateral boundaries are the left main bronchus and the extended line of the left margin of the trachea. | 109 L |  |
| M |  | Middle paraoesophageal | Lymph nodes located around the middle thoracic esophagus | 108 | 8M |
| M |  | Subcarnial | Lymph nodes located caudal to the carina of the trachea. The lateral boundaries are the extended line of both lateral margins of the trachea | 107 | 7 |
| M |  | Right recurrent nerve | Lymph nodes located along the right recurrent laryngeal nerve | 106recR | 2R |
| M |  | Right tracheobronchial | Right tracheobronchial lymph nodes: The superior border is the inferior wall of the azygos vein | 106tbR | 4R |
| O |  | Left recurrent nerve | Lymph nodes located along the left recurrent laryngeal nerve | 106recL | 2L |
| O |  | Left tracheobronchial | Left tracheobronchial lymph nodes: The superior border is the inferior wall of the aortic arch, and the lymph nodes are located in the area surrounded by the medial wall of the aortic arch | 106tbL | 4L |

TTE = transthoracic esophagectomy, THG = transhiatal extended gastrectomy, M = mandatory to resect, O = optional to resect, JGCA Japanese Gastric Cancer Association, JES Japan Esophageal Society, AJCC American Joint Committee of Cancer, UICC Union for International Cancer Control.
